# High-Throughput Analysis of Promoter Occupancy Reveals New Targets for *Arx*, a Gene Mutated in Mental Retardation and Interneuronopathies

**DOI:** 10.1371/journal.pone.0025181

**Published:** 2011-09-22

**Authors:** Marie-Lise Quillé, Solenne Carat, Sylvia Quéméner-Redon, Edouard Hirchaud, Daniel Baron, Caroline Benech, Jeanne Guihot, Morgane Placet, Olivier Mignen, Claude Férec, Rémi Houlgatte, Gaëlle Friocourt

**Affiliations:** 1 Inserm U613, Brest, France; 2 Faculté de Médecine et des Sciences de la Santé, Brest University, Brest, France; 3 Laboratory of Molecular Genetics and Histocompatibility, Brest University Hospital, Brest, France; 4 Institut du Thorax, Inserm U915, Nantes University, Nantes, France; 5 Etablissement Français du Sang (EFS) Bretagne, Brest, France; Stanford University, United States of America

## Abstract

Genetic investigations of X-linked intellectual disabilities have implicated the *ARX* (*Aristaless-related homeobox*) gene in a wide spectrum of disorders extending from phenotypes characterised by severe neuronal migration defects such as lissencephaly, to mild or moderate forms of mental retardation without apparent brain abnormalities but with associated features of dystonia and epilepsy. Analysis of *Arx* spatio-temporal localisation profile in mouse revealed expression in telencephalic structures, mainly restricted to populations of GABAergic neurons at all stages of development. Furthermore, studies of the effects of *ARX* loss of function in humans and animal models revealed varying defects, suggesting multiple roles of this gene during brain development. However, to date, little is known about how ARX functions as a transcription factor and the nature of its targets. To better understand its role, we combined chromatin immunoprecipitation and mRNA expression with microarray analysis and identified a total of 1006 gene promoters bound by Arx in transfected neuroblastoma (N2a) cells and in mouse embryonic brain. Approximately 24% of Arx-bound genes were found to show expression changes following Arx overexpression or knock-down. Several of the Arx target genes we identified are known to be important for a variety of functions in brain development and some of them suggest new functions for Arx. Overall, these results identified multiple new candidate targets for Arx and should help to better understand the pathophysiological mechanisms of intellectual disability and epilepsy associated with *ARX* mutations.

## Introduction

Intellectual disability (ID) is usually defined as a heterogeneous group of disorders resulting from a variety of acquired and genetic causes, and is thought to affect approximately 1% of the population across the world [1]. The observation that ID is preferentially expressed in males has led to focus on genes located on the X chromosome. To date, more than 90 associated genes have been identified [2]. One of the most frequently mutated genes is the *Aristaless-related homeobox* (*ARX*) gene (Entrez Gene 170302) located on Xp22.13. It was first identified in 2002 as involved in non-syndromic X-linked mental retardation (OMIM 300382 and 300419) [3] and West syndrome (OMIM 308350) [4], [5]. Since then, several other mutations have been described and implicated *ARX* in a wide spectrum of disorders extending from phenotypes with severe neuronal migration defects such as X-linked lissencephaly with abnormal genitalia (XLAG), to milder forms of mental retardation without apparent brain abnormalities but with associated features of dystonia and epilepsy [3]–[10].

Although Arx is expressed in several structures including the brain, pancreas, developing testes, heart, skeletal muscle and liver [3], [6], [11]–[13], the most striking consequences of *Arx* loss of function concern the brain and testis in both mouse and human. In the developing and adult brain, Arx is strongly expressed in telencephalic structures, particularly in populations of GABA-containing neurons [15]–[17]. Recent studies of the effects of *Arx* loss of function revealed that this gene contributes to almost all fundamental processes of brain development: patterning, neuronal proliferation and migration, neuronal maturation and differentiation as well as axonal outgrowth [6], [14], [17], [18].

*Arx* encodes a transcription factor which belongs to one of the three largest classes of homeoproteins, the paired (Prd) class. Genes from this family are characterised by a 60-amino acid homeodomain responsible for DNA-binding. In addition, they often contain other motifs that can contribute to DNA and/or co-factor binding specificity. For example, the highly conserved octapeptide domain located in the N-terminal part of Arx, as well as another C-terminal region including the fourth polyalanine tract, have transcriptional repressor activity while the aristaless-related domain, located at the C-terminus, has transcriptional activator activity [19], [20]. A few co-factors of Arx have been identified: the Groucho/transducin-like enhancer of split (TLE) family of co-repressors interacts with Arx octapeptide, whereas repression by the second domain occurs through the interaction with C-terminal binding proteins (CtBPs) [20]. In addition, Arx has four polyalanine tracts. The two ones located in the N-terminal part of Arx seem particularly important for the function of the protein as several expansions have been identified in patients. For this type of mutations, it has recently been suggested that the level of transcriptional repression activity may depend on the length of the alanine expansion [19]. Changes in the transcriptional activity of Arx may thus have subtle effects on neuronal function and contribute to the pathogenesis of *ARX*-related disorders, in particular intellectual deficiency and epilepsy.

Although two gene expression profile analyses comparing E14.5 wild-type and *Arx* mutant ventral telencephalic tissues have recently been published in mouse, very few targets for this transcription factor have been described and only three (*Lmo1*, *Ebf3* and *Shox2*) have been found to be direct [21], [22]. Here, using chromatin immunoprecipitation in Arx-transfected neuroblastoma cells (N2a) or E15.5 mouse embryonic brain, followed by hybridization to mouse promoter arrays (ChIP-chip) [23], we identified new direct targets of Arx. We found a total of 1006 Arx-bound genes. A significant proportion of these promoters were enriched for a sequence very similar to a motif previously identified as Arx-binding motif. Both fixation and regulation of subsets of these targets have then been confirmed by ChIP-PCR and by the analysis of transcriptomic experiments performed from either Arx-overexpressing N2a cells or *Arx* knock-out mice.

## Results

### Assessment of the specificity of the ChIP protocol

**Figure 1:**
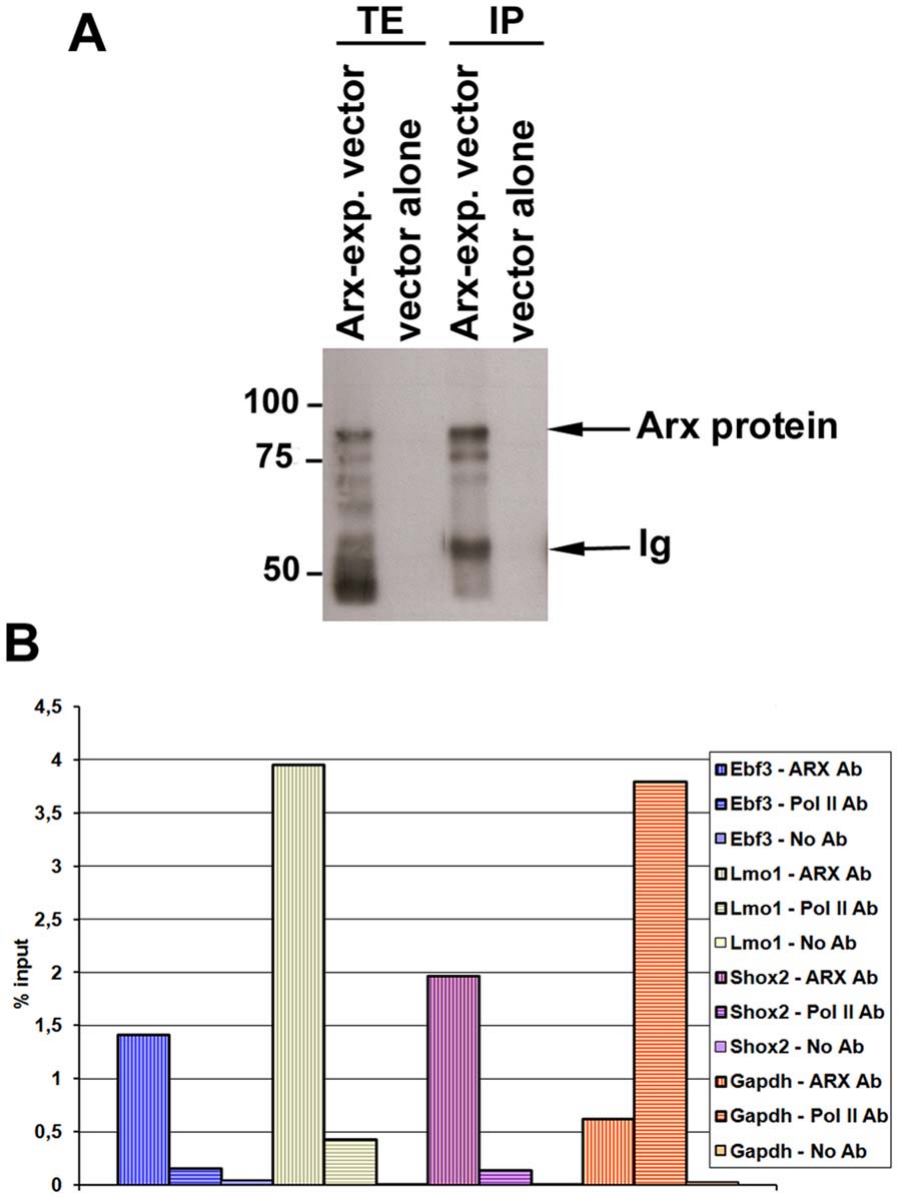
Assessment of the specificity of the ChIP protocol. A) Arx immunoprecipitation from N2a cells transfected with an Arx-expressing vector or a vector alone and using the Arx C-14 antibody from Santa Cruz. Detection using the anti-Arx-HD antibody revealed a major band at approximately 80 kD, which corresponds to the size of Arx protein. TE: total extracts, IP: immunoprecipitation, Ig: immunoglobulins. B) Enrichment of *Ebf3*, *Lmo1* and *Shox2* promoter regions was assessed by qPCR using either Arx or PolII-immunoprecipitates or using no antibody and was compared to the total input. *Gapdh* was used as a positive control with DNA Pol II. Here, we show an example of the results obtained in a ChIP experiment with Arx C-14 antibody from Santa Cruz. The enrichment of *Ebf3*, *Lmo1* and *Shox2* was checked similarly for each replicate experiment before DNA was applied to the microarrays.

To better understand the role of *Arx* and the effect of its mutations on brain development, we used chromatin immunoprecipitation on promoter arrays (ChIP-chip) to identify direct targets of this transcription factor. As Arx is not endogenously expressed in any known neuronal cell line, we decided to use Arx-transfected mouse neuroblastoma (N2a) cells. This cell line has recently been used to validate three direct targets of Arx (*Lmo1*, *Ebf3* and *Shox2*) [21]. We first tested the ability of two different polyclonal Arx antibodies (anti-Arx-HD directed against the homeodomain region of Arx and produced by Poirier et al. [15] and Arx C-14 from Santa Cruz directed against the C-terminal part of the protein) to work in protein immunoprecipitation. Both antibodies could immunoprecipitate the transfected Arx protein, however the antibody from Santa Cruz seemed a bit more efficient than anti-Arx-HD (data not shown and Figure 1A). To test the ability of these antibodies to work in chromatin immunoprecipitation, we took advantage of the previous identification of three direct targets of Arx [21]. N2a cells transfected with Arx were first treated with formaldehyde to preserve interactions between Arx and its target DNA. Following cell lysis, genomic DNA was sheared into fragments of 300–600 bp and precipitated using i) one of the two polyclonal Arx antibodies, ii) an antibody directed against DNA Polymerase II which is known to bind the *Gapdh* gene, iii) without any antibody. Then, the presence of *Lmo1*, *Ebf3* and *Shox2* in each immunoprecipitate was assessed by quantitative PCR (qPCR) (Figure 1B). The results showed that Arx C-14 antibody yielded successful enrichment of the 3 specific targets which, on the opposite, were not present in the anti-PolII immunoprecipitates (Figure 1B). Similar results were obtained with anti-Arx-HD although this antibody seemed slightly less specific (data not shown). We thus decided to use the commercial antibody in most of the following experiments. We then amplified, labelled and cohybridized Arx-immunoprecipitated DNA and total DNA input onto Agilent mouse promoter arrays.

### Identification of an Arx-binding motif in ChIP-positive sequences obtained from Arx-transfected N2a cells

**Figure 2:**
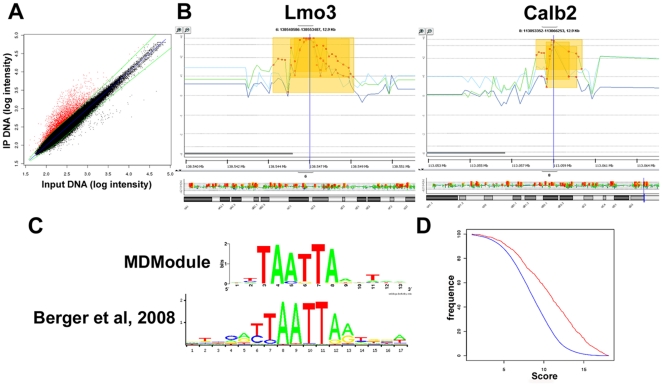
ChIP-chip results obtained from Arx-transfected N2a cells. A) Graph representing log_2_ probe intensities of Arx-immunoprecipitated DNA (IP) and input DNA obtained in a representative ChIP experiment. The red dots indicate the probes enriched in Arx-immunoprecipitates compared to total input DNA. B) Examples of the enrichment profiles of Arx-bound promoter regions visualized by DNA analytics software. The 3 lines represent data obtained from 3 independent ChIP-chip experiments and show the reproducibility between the 3 replicates. C) The resulting weighted matrix discovered through the MDModule analysis (top) appears to be similar to the motif identified by Berger et al. [27] (bottom). D) Frequency distribution of scores. The TAATTA motif identified by MDModule was significantly more present in ChIP-enriched sequences (red curve) by comparison to negative control sequences (blue curve).

The mouse promoter microarrays from Agilent Technologies (G4490A) consist of two arrays with approximately 244 000 60-mer oligonucleotide probes spaced every 100–300 bp across regions of interest. They cover −5.5 kb upstream to +2.5 kb downstream of the transcriptional start sites of approximately 17,000 of the best-defined mouse genes. These arrays have previously been used to identify binding sites of other important transcription factors such as Pax6 [24]. Three independent ChIP-chip experiments were performed from N2a cells transfected with Arx. As previously described (Figure 1B), the enrichment of *Lmo1*, *Ebf3* and *Shox2* was checked in each replicate before immunoprecipitated DNA was applied to the microarrays. These experiments identified 927 genes consistently enriched in Arx-immunoprecipitated material (geometrical mean of the *P*-values of the 3 experiments ≤10^−4^ with a *P*-value<10^−3^ in at least 2 experiments) (Table S1) (Figure 2A–B).

To investigate whether target genes contain a consensus motif that could correspond to Arx-binding motif, we took the 500 most enriched genes based on *P*-values (Table S1) and used an unbiased approach with the MDModule of the MotifRegressor program [25] to identify sequence motifs enriched within 450 bp of the center of the corresponding positive probes. This approach detected several motifs in a significant number of Arx-immunoprecipitated probes. Similar motifs were then gathered using the MotifsComparator program [26] to lead to a consensus sequence, which appeared to be similar to the one defined by Berger et al. [27] as Arx-binding motif (Figure 2C). These authors identified this motif by using protein-binding microarrays to determine the *in vitro* DNA-binding preferences of several mouse homeodomains to all possible 8-nucleotide sequences [27]. In our experiments, the Arx-immunoprecipitated promoters were unambiguously enriched for this motif by comparison to control (*P*-value not significant in any replicate) promoter regions not bound by Arx (Figure 2D). Next, we used the EMBOSS Profit algorithm to search all ChIP-positive probes using both motifs. We found that 490 genes (≈53%) of promoter regions bound by Arx in all three experiments contained one or several motifs with at least 75% similarity to Arx-binding motif. This result was significantly higher than the number of motifs obtained in a set of control (probes with a *P*-value not significant in any replicate) sequences.

### Identification of Arx targets from mouse embryonic brains

**Figure 3:**
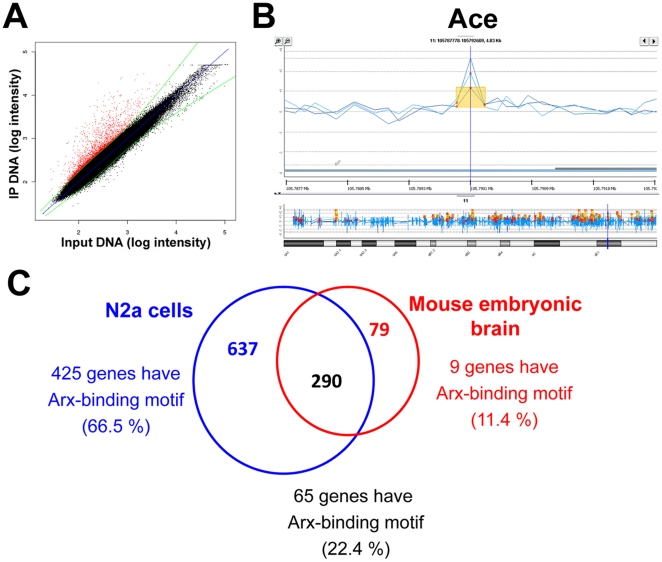
ChIP-chip results obtained from mouse embryonic brain. A) Graph representing log_2_ probe intensities of Arx-immunoprecipitated DNA (IP) and input DNA obtained in a representative ChIP experiment. The red dots indicate the probes enriched in Arx-immunoprecipitates compared to total input DNA. B) Example of the enrichment profiles of Arx-bound promoter regions visualized by DNA analytics. The 3 lines represent data obtained from 3 independent ChIP-chip experiments and show the reproducibility between the 3 replicates. Contrarily to N2a cells, in which several continuous probes were often found enriched, only one or two probes were found enriched per gene. C) Venn diagram illustrating the overlap (black) between Arx-immunoprecipitated genes in transfected N2a cells (blue) and mouse embryonic brain (red), and the number of genes with at least 75% match to Arx-binding motif.

To validate these results in a more physiological situation, we decided to do similar experiments from E15.5 mouse embryonic brains, which correspond to an important time for neuronal migration and differentiation and which shows a high expression of Arx. We thus performed 3 independent experiments, hybridizing Arx-associated chromatin fragments to the same promoter microarrays. In total, 369 genes were found consistently enriched in Arx-immunoprecipitated material (geometrical mean of the *P*-values of the 3 experiments ≤10^−3^ with a *P*-value<10^−3^ in at least 2 experiments) (Table S1) (Figure 3A–B). Our results revealed that out of these 369 genes, 290 were common to those identified in transfected N2a cells (Figure 3C).

Then, using EMBOSS Profit as previously, we inspected the sequences identified in embryonic brain for both Arx-binding motifs (Figure 2C). Surprisingly, out of 369 genes, only 74 (20%) were found to have at least one Arx-binding site as previously defined (Figure 3C). Among these 74 genes, 65 genes were identified in both Arx-transfected N2a cells and mouse embryonic brain (Figure 3C). Although we tried to identify new or degenerated motifs in these negative sequences, we were not able to find a motif that was significantly more present in Arx-bound sequences by comparison to control (probes with a *P*-value not significant in any replicate) sequences.

### Validation of the fixation of Arx

**Figure 4:**
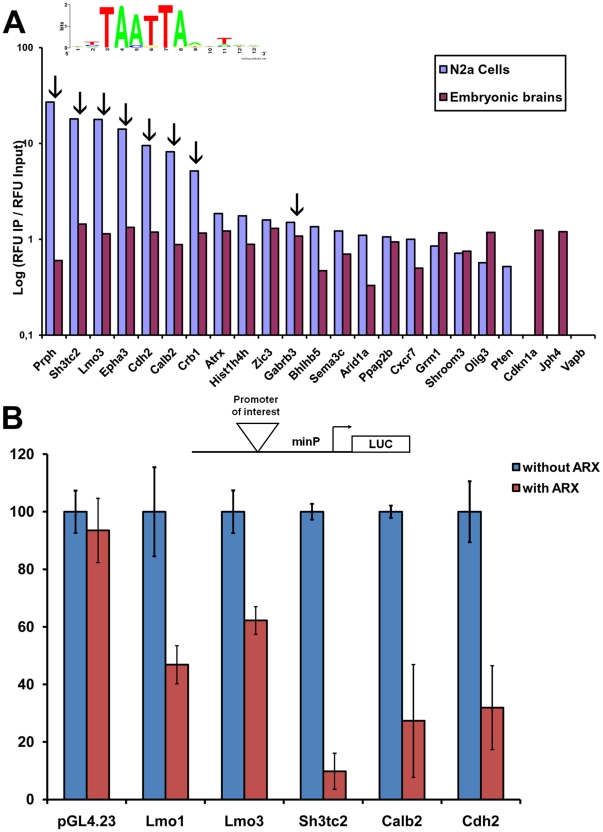
Confirmation of Arx binding to candidate promoter regions. A) Arx-immunoprecipitated DNA was compared to input DNA by ChIP/QFM-PCR to determine ChIP enrichment of 21 putative target genes. No enrichment occurred for the *Vapb* promoter which was consistently negative in all ChIP experiments. Bar heights represent log_10_ enrichment of the signal obtained for Arx-immunoprecipitated DNA versus input DNA for each promoter using site-specific primers. The arrows above the bars indicate sequences in which the previously defined Arx-binding motif was found. B) Confirmation of Arx binding to 5 different promoter sequences identified by ChIP using a luciferase reporter gene assay. *Firefly* luciferase data were normalized to *Renilla* luciferase expression and data are presented as the percentage of transcriptional activity compared to the vector control. Arx regulation was confirmed for *Lmo1*, *Lmo3*, *Sh3tc2*, *Calb2* and *Cdh2* promoter regions as transcriptional activity was repressed in the presence of Arx by comparison to the control transfection, whereas it had no effect on the plasmid alone pGL4.23. Error bars indicate SEM.

To determine the validity of our ChIP-chip results, we randomly selected 21 candidate Arx-bound genes displaying representative degrees of enrichment based on *P*-values in the list of 1006 genes obtained in total in ChIP experiments (Figure 3C, Table S1). We performed quantitative QFM-PCR on Arx-immunoprecipitated material from transfected N2a cells and embryonic brain and compared the enrichment of these genes with total input DNA. Binding was confirmed for 19/21 genes in both transfected N2a cells and embryonic brain (Figure 4A). In contrast, there was no enrichment of any of these genes in control immunoprecipitates (anti-PolII or without any antibody) (data not shown). Similarly, Arx did not bind to *Vapb*, a negative control (*P*-value not significant in any experiment) (Figure 4A). These results confirmed ChIP-chip findings for *Pten*, which was only identified in N2a cells on microarrays and was also found negative in embryonic brain by ChIP-PCR. Similarly, *Jph4* which was hardly positive in N2a cells by ChIP-chip was only confirmed in brain by ChIP-PCR (Figure 4A). However, we observed that we were able to validate in both Arx-transfected N2a cells and E15.5 embryonic brain some genes, such as *Sh3tc2*, *Lmo3*, *Epha3*, *Cdh2*, *Calb2* that were negative in ChIP experiments performed from embryonic brains, suggesting a higher sensitivy of the quantitative ChIP-PCR method compared to ChIP-chip, at least in embryonic brains. Taken together, these observations confirm the specificity of our ChIP-chip results and that Arx not only binds *in vivo* to some of the 290 common genes obtained in N2a cells and mouse embryonic brain (as shown in Figure 4A for *Prph*, *Atrx*, *Hist1h4h*, *Gabrb3*, *Bhlhb5*, *Sema3c*, *Arid1a*, *Ppap2b*, *Cxcr7*, *Grm1*, *Shroom3* and *Olig3*), but also to genes that were identified only in N2a transfected cells (such as *Sh3tc2*, *Lmo3*, *Epha3*, *Cdh2*, *Calb2*, *Crb1*).

In addition, we observed that although the degree of enrichment of candidate genes containing the TAATTA motif (indicated by the arrows on Figure 4A) tended to be higher in Arx-immunoprecipitates from N2a cells, there appeared to be no correlation between the enrichment and the presence of the motif in genes immunoprecipitated from embryonic brain (Figure 4A). These results thus suggest that whereas overexpressed Arx seems to be primarily recruited to target genes by direct association with the previously defined motif, in a more physiological situation such as in embryonic brain, Arx is either recruited by association to other less common motifs that were not identified by MDModule or may be recruited through interaction with other cofactors. This hypothesis may explain why *Sh3tc2*, *Lmo3*, *Epha3*, *Cdh2*, *Calb2*, *Crb1* genes were found to have such a high *P*-value in ChIP-chip experiments from transfected N2a cells but not in mouse embryonic brain although the interaction also exists in embryonic brain as shown by ChIP-PCR on Figure 4A.

To provide independent validation of these results, we also tested *Lmo1*, *Lmo3*, *Sh3tc2*, *Cdh2* and *Calb2* using a luciferase reporter approach and measured the level of transcriptional activity after Arx transfection into N2a cells. All five genes displayed repressed transcriptional activity following transfection with Arx, contrarily to the *Firefly* luciferase plasmid pGL4.23 alone (Figure 4B).

### Functional annotations of Arx-bound genes

**Figure 5:**
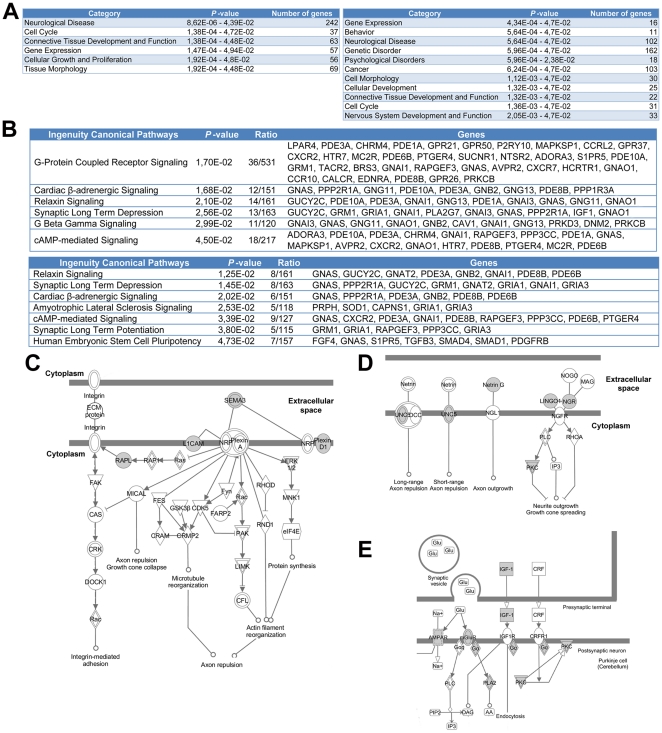
Functional annotations of Arx targets. A) Significantly enriched biological functions obtained from the data in transfected N2a cells (left) or embryonic brain (right) confirm already known functions of Arx in the regulation of gene expression, cellular growth and proliferation and emphasizes its important role in the development of neurological and psychological disorders. The indicated *P*-value was calculated by Fisher's exact test. B) Significantly enriched canonical pathways associated with Arx targets. The top table indicates enriched genes from transfected N2a cells and the bottom one indicates results from embryonic brain. The indicated *P*-value was calculated by Fisher's exact test. Ratios correspond to the number of ChIP-positive genes compared to the genes present on the array for each canonical pathway. C) and D) Examples of ChIP-positive genes (indicated in grey) playing a role in the Axonal Guidance canonical pathway (image generated by the IPA program). E) Example of ChIP-positive genes (indicated in grey) playing a role in synaptic transmission (image generated by the IPA program).

To identify the cellular processes regulated by Arx, we examined the functional annotations associated with ChIP-positive genes, based on the Gene Ontology (GO) database [28]. Using DAVID and Ingenuity Pathway Analysis programs, we identified several enriched functions important for brain development and potentially linked to neurological and/or psychological disorders (Figure 5A). Data from transfected N2a cells revealed an enrichment of several biological functions already known to be associated with Arx, such as the regulation of cell cycle, gene expression, cellular growth and proliferation (Figure 5A, left). Data obtained from mouse embryonic brains showed, in addition, a significant enrichment of genes that were found to be important in mice behavior studies (Figure 5A, right). At the cellular level, Arx-bound promoters are enriched for genes important for nuclear transport, cell adhesion or migration, RNA processing, cell fate commitment and regulation of multicellular process through the regulation of several important signaling pathways including G-protein coupled-receptor, adenylate cyclase activity or neuroactive ligand-receptor interaction (Figure 5B). Interestingly, several genes identified in N2a-transfected cells and embryonic brain were associated with axonal guidance (Figure 5C–D) and long-term depression and/or long-term potentiation, suggesting a possible function of Arx in synaptic plasticity (Figure 5E). We also examined in more detail Arx targets associated with neuronal function. As shown in Table 1, a certain number of them have been associated with dyskinesia and neurological disorders including phenotypes observed in *ARX*-mutated patients.

**Table 1:** Examples of Arx-immunoprecipitated genes and their corresponding functional category.

Function	Molecules
schizophrenia	AHI1, ALK, BRD1, FXYD6, GNAS, GRIA1, HTR7, LGI1, NAALAD2, NDE1, NTNG1, PPP3CC, SIGMAR1, SLC1A1, TH, TPH1, NRXN1, BRD1, CCDC60
mental retardation	ATRX, GRIA3, PRSS12, TSPAN7, AHI1, TIMM8A, BBS12, ATRNL1, ZDHHC9, NRXN1, L1CAM, RAB39B, RASGEF1B, ALG2, NFIB, SNRPN
epilepsy	GABRB3, GABRE, GAD2, GRIA1, GRIA3, LGI1, OTX1, SLC12A5, LGI1, OTX1, KCNAB1, KCNJ14, RAB39B
autism	AHI1, ATRX, BTD, FGFGR2, GRIA3, L1CAM, NRXN1, PRSS12, PTEN, RAB39B, NTNG1, NRXN1
dyskinesia	ACTN2, AEBP1, ARMCX2, ATP5J, CAPNS1, CASQ1, CCKBR, CDH2, CHN1, CHRM4, CRB1, CREB3L1, CYP26B1, EGR1, EPHX2, FOSB, GABRB3, GABRE, GAD2, GNAO1, GNAT2, GPD1, GRIA1, GRIA3, HBP1, HSPA5, IDO1, IGF1, KCNA5, KCNAB1, MEIS2, MYT1L, NDRG1, NKX2-1, NR4A1, OXR1, PDE10A, PDE6B, PDLIM1, PFN2, PPARG, PPARGC1A, PPP1R3A, PRKCB, PRL, RAB5A, SCOC, SDC4, SLC1A1, SLC6A2, SLIT2, SPOCK3, SRPX, TCERG1, TH, TPD52, TSC22D3, TUBA1A, UGCG
depressive disorder	ACE, ATG5, BTD, CCKBR, CHGA, CHRM4, CHRNG, CTR9, GABRB3, GABRE, GPR50, GRIA1, GRIA3, HTR7, IGF1, LMNB2, MDFI, PDE10A, PDE1A, PPARG, SIGMAR1, SLC1A1, SLC6A2, TACR2, TOB1, TPH1, WFS1
synaptic plasticity and synaptogenesis	GHRH, MUSK, PCDHB5, PCDHB10, DNM2, P2RX1, GLRA3, PRSS12, GABRB3, GRIA3, DOC2B, CHRNG, CHRM4, GRM1, PSD3, SV2A, CPEB1, LIN7C, L1CAM, GRIA1, GABRE, TH, NRXN1, GAD2, DGKZ, LRRTM2, ACCN5, ACTN2, ACTR3, ACVRL1, ADORA3, ARHGAP5, CACNA2D1, CADM3, CAV1, CCKBR, CDH2, CNTN1, CTNNA2, CYBB, NRXN1, LGI1, CACNG4, CTNNA2, RASGEF1B, CDH2, JPH4, RTN4R, SLC1A1, EPHB1, PPP2R1A, GNAI3, GNAO1, GNAI1, IGF1, GNAS, GRIA3, GRM1, EGR1
neuronal differentiation	TH, L1CAM, PTEN, EPHB1, OTP, MYCBP2, LINGO1, SRPX, CRB1, POU4F3, POU4F2, LHX5, PHOX2A, EFNB3, FOXA1, NTNG1, SMAD4, NR4A2, RTN4R, SOD1, FGF20, SLIT2, GNAT2, FOXN4, CTNNA2, SALL3, SEMA6A, NRL
axonogenesis	SEMA6A, EFNB3, POU4F3, NTNG1, RTN4R, NR4A2, POU4F2, L1CAM, EPHB1, SLIT2, CTNNA2, MYCBP2, CHD2, SHROOM3, EDNRA, CHN1
axonal guidance	GNAI3, EFNB3, GNAI1, NTNG1, L1CAM, SLIT2, EPHB1, EPHA3, SEMA6A, SEMA3G, PPP3CC, SEMA3C, UNC5D
size and morphology of the brain	HSF2, L1CAM, PTEN, IGF1, NDE1, PTEN, WASF1, CTNNA2, AHI1, ASPM, ARHGAP5, CDH2, TUBA1A, EOMES

Using publicly available *in situ* hybridization data in mouse (GenePaint, [29]), we next investigated the expression of some Arx candidate target genes (Figure S1). In the developing cortex, Arx has been shown to be expressed in progenitors of the dorsal ventricular and subventricular zones as well as in tangentially migrating interneurons coming from the ganglionic eminences but not in radially migrating cells. On the contrary, in the basal telencephalon, Arx is strongly expressed in differentiated neurons but not in proliferating cells of the lateral and medial ganglionic eminences [15]–[17]. As shown in Figure S1, several ChIP-positive genes are expressed in the developing telencephalon at E14.5, either in the cortical ventricular zone, or in regions containing migrating and/or differentiating interneurons. For example, at E14.5, *Epha3*, *Sema3c*, *Cxcr7* and *Snrpn* appear to be expressed in cortical migrating neurons, whereas *Bhlhb5* and *Gabrb3* seem to be restricted to differentiating neurons in the cortical plate. Genes such as *Cdh2*, *Rpn1*, *Nxf1* and *Tle1* appear to be also expressed in cortical neuronal progenitors. Finally, genes like *Tle1*, *Hist1h4h*, *Hist2h3c*, *Rpn1* and *Nxf1* also localize to developing ganglionic eminences suggesting a possible role in interneuron development (Figure S1).

Several ChIP-positive genes also have a role in the development of other tissues in which Arx has been shown to be important, such as the pancreas (*Arntl*, *Onecut1*, *Brd2*, *Cdx1*, *Creb3l1*, *Dusp19*, *Pik3r3*…) or skeletal muscle (*Casq1*, *Musk*, *Myh8*, *Ank1*, *Meg3*…). Others have important function in testis development (*Cstf2t*, *Fert2*, *Phkg2*, *Pxt1*, *Alkbh2*, *Ankrd7*…). Further investigations of the functions of Arx-potential targets also suggest that Arx may play a role in a number of other processes in which it had not been previously implicated such as in heart and blood vessel development, bone formation and osteoblast differentiation, proliferation and differentiation of myeloid/mesenchymal cells, stem cell maintenance, RNA processing, protein transport and clathrin-mediated endocytosis (Tables S2 and S3). Taken together, these results suggest that Arx may be involved in a wide range of cellular pathways at the transcriptional level.

### Gene expression analysis of Arx-overexpressing N2a cells

**Figure 6:**
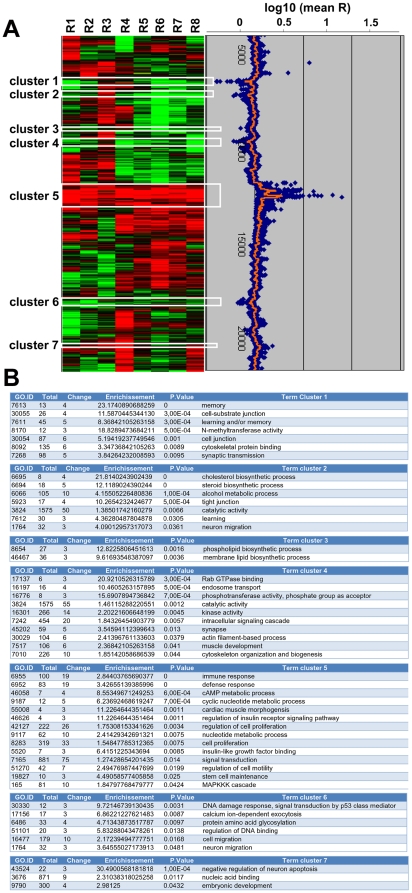
Gene expression analysis following Arx expression in N2a cells. A) Heat map representing hierarchical clustering of the ratio “Arx-transfected cells/control-transfected cells” in 8 different replicates. Each row represents a gene and each column, a sample. Levels are color-coded with red, green and black, corresponding to an increase, decrease or no change in gene expression, respectively. Seven clusters of genes with the same regulation were identified. B) Functional annotation of genes in the seven clusters using the GoMiner program.

As binding does not necessarily imply regulation, we next checked whether the putative targets identified by ChIP-chip were differently regulated following Arx overexpression. We thus compared gene expression between Arx-transfected N2a cells and cells transfected by the corresponding empty vector. We did 8 different independent experiments on which qRT-PCR was first performed to confirm the expression of *Arx* in samples transfected with Arx (data not shown). Then, cRNAs were hybridized on microarrays representing 39430 Entrez Gene RNAs (Agilent Technologies). Following hierarchical centroid-linkage clustering, differentially expressed genes were grouped according to similar variation in gene expression patterns, resulting in the identification of 7 different clusters that represent 2145 genes (Figure 6A). Among them, 908 genes (43%) were up-regulated and 1237 (59%) were down-regulated in Arx-expressing cells, confirming previous observations indicating that Arx acts primarily as a transcriptional repressor [20], [21], [30]. As a confirmation of these results, we noted that several of the genes we identified in the 7 clusters such as *Lmo1*, *Lmo4*, *Etv1*, *Lonrf3*, *Il17rd*, *Pcdh17*, *Socs2*, *Ank3*, *Atp7a*, *Bmper*, *Foxp1*, *Phlda1*, *Zfp503* and *Vcan* had previously been found deregulated in the striatum of *Arx* knock-out mice [21], [22].

To investigate whether identified genes belong to functional categories which could provide further insight into the role of Arx, we used GoMiner to look at GO categories containing at least 3 genes and having a *P*-value≤0.05 by Fisher's exact test. This analysis revealed that genes involved in learning and/or memory function or synaptic transmission were found to be down-regulated following Arx ectopic expression (clusters 1–4 and 6), whereas those involved in signal transduction or cell proliferation were up-regulated (clusters 5 and 7) (Figure 6B).

**Figure 7:**
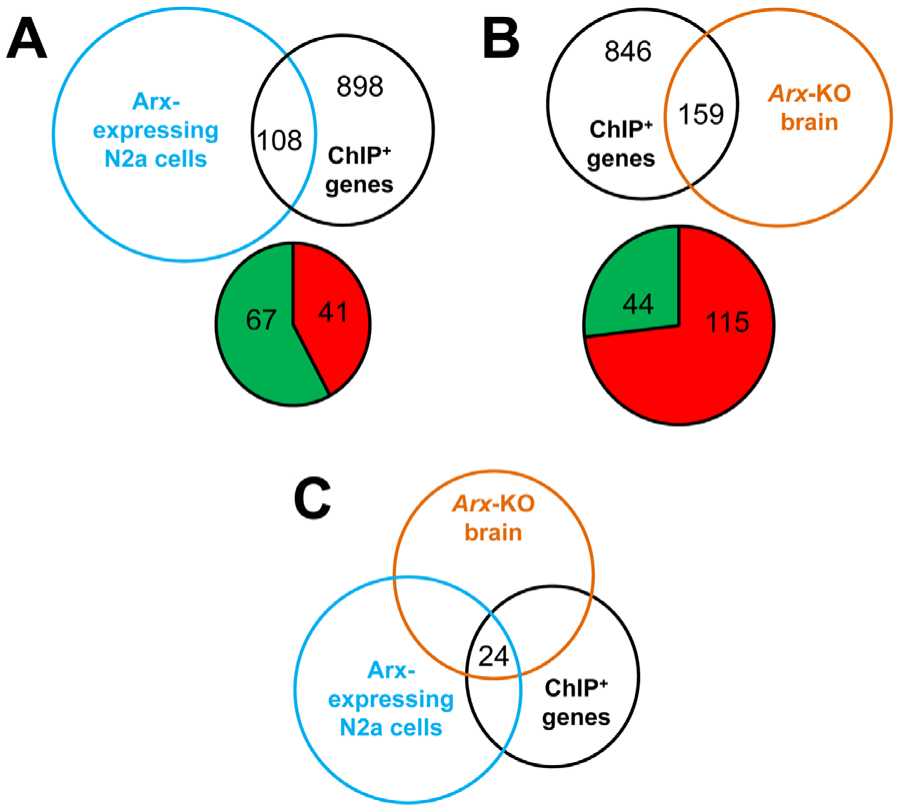
Gene expression changes of Arx candidate targets. A) Venn diagram illustrating the number of ChIP-positive genes that were found to show expression changes in Arx-overexpressing cells. The Pie diagram shows the number of ChIP-positive genes that are down-regulated (indicated in green) or up-regulated (indicated in red). B) Venn diagram illustrating the number of ChIP-positive genes that were found to show expression changes in *Arx* knock-out ventral forebrain. The Pie diagram shows the number of ChIP-positive genes that are down-regulated (indicated in green) or up-regulated (indicated in red). C) Venn diagram illustrating the number of ChIP-positive genes that show gene expression changes in both Arx-overexpressing cells and *Arx* knock-out mice.

We next compared the list of the 927 ChIP-positive sequences obtained in transfected N2a cells (Figure 3C, Table S1) with the genes identified in the 7 clusters. Among genes represented on both the expression and promoter microarrays, we found that 95 of the 927 Arx-bound genes (≈10.2%) displayed expression changes when Arx was overexpressed in N2a cells. This percentage increased to 10.7% (108 genes out of 1006) when we included the 79 genes that were found only in ChIP experiments from embryonic brains (Figure 7A, Table S4).

### Gene expression analysis of *Arx* knock-out mice

To investigate whether binding of Arx to candidate targets have detectable effects on gene expression in more physiological conditions, we took advantage of two studies comparing gene expression between basal telencephalon of E14.5 *Arx* knock-out and wild type mice [21], [22]. These authors respectively identified 38 [22] and 84 genes [21], which expression was deregulated in *Arx* knock-out mice. However, as both studies focused on genes having a Fold-Change superior to 2 and our aim is to validate potential targets of Arx which display changes in gene expression but not necessarily important ones, we used the GeneSpring software from Agilent Technologies to reanalyze their data. We identified 2537 genes with expression changes >1.1 fold (*P*-value≤0.05) between *Arx*-mutated brains and controls. Taking into account the two lists of published genes [21], [22] and these data, we observed that out of the 369 Arx putative targets identified from mouse embryonic brain, 61 genes (≈16.5%) showed significantly changes in gene expression in *Arx*-mutant brains. This percentage changed to 15.8% (159 genes out of 1006) when we included the 637 genes that were found only in ChIP experiments from Arx-transfected N2a cells (Figure 7B, Table S4).

Arx was previously described to act as both a transcriptional repressor and transcriptional activator [20], [21], [30]. We thus looked at whether Arx regulates genes in the same way in transfected N2a cells and mutant brain. We noticed that the majority (67/108) of Arx-bound genes that showed gene expression changes in transfected cells were down-regulated (Figure 7A). On the opposite, the majority (115/159) of Arx-bound genes that showed gene expression changes in *Arx* knock-out mice was up-regulated (Figure 7B). This result is consistent with previous studies suggesting that Arx acts primarily as a transcriptional repressor [20], [21], [30]. Interestingly, when looking at the 24 ChIP-positive genes that show gene expression changes in both Arx-overexpressing cells and *Arx* knock-out mice (Figure 7C), we noticed that 9 genes (Table S4) showed the same type of regulation when Arx was overexpressed or down-regulated (as for example *Phlda1*) whereas 15 genes (Table S4) showed opposite behavior (as for example *Kcna5*), suggesting that depending of the biological context or the molecular environment, Arx may activate or repress the same genes. We also noticed that there was no correlation between the presence of the previously identified Arx-binding site and the deregulation of these genes (data not shown), suggesting once again that the TAATTA motif does not necessarily mean regulation and that Arx may bind to other DNA motifs. Taken together, our results demonstrate that a substantial proportion of the direct targets of Arx identified by ChIP-chip show gene expression changes following Arx overexpression or knock-down.

## Discussion

*ARX* loss-of-function mutations in mouse and human have been shown to affect several parts of the brain including the cortex, thalamus, hippocampus, striatum and olfactory bulb [14], [31], [32]. In particular, Arx has been shown to be important for both radial and tangential migration and interneuron subpopulation differentiation [6], [14], [18]. Given the different roles Arx has been suggested to play in brain development, it would be now interesting to uncover the cellular pathways controlling these processes by identifying the downstream targets of Arx in the central nervous system. Therefore, in this work, we combined ChIP method to identify Arx-bound promoters with studies of gene expression changes following Arx overexpression or knock-down.

In total, we identified 1006 putative Arx-bound genes, several of them involved in the patterning of the central nervous system, axonal guidance, neurodevelopment, neurotransmission and neurite outgrowth. Interestingly, we also suggest new possible functions for Arx, as for example in osteoblast differentiation or mesenchymal cell proliferation and differentiation. Although these functions may be surprising as first glance, they may be related to the expression of Arx in the neuroepithelium [15]–[17] and the recent finding that mesenchymal stem cells are generated early in development from the neuroepithelium [33]. As a validation of our results, we identified in our ChIP-chip experiments *Lmo1* and *Ebf3* which had previously been described as direct targets of Arx [21]. In addition, the use of a different Arx antibody (anti-Arx-HD) which identified a large number of overlapping targets in ChIP experiments (data not shown), provides an independent level of confirmation of our results.

Comparison between ChIP experiments performed in transfected N2a cells and mouse embryonic brain revealed a large degree of overlaping identified targets. In particular, we observed that most of the genes identified only in embryonic brain were also enriched in transfected cells, although the enrichment was below our threshold of selection (see *P*-values in Table S1). For the vast majority of the genes that we tested by ChIP-PCR, we were able to validate Arx promoter binding not only in transfected N2a cells but also using mouse embryonic brains immunoprecipitates (Figure 4A). Similarly, we confirmed Arx fixation to *Zic3*, and yet this gene was below our threshold of selection as it was positive in only one experiment in transfected cells and one experiment in embryonic brain. This observation suggests that some genes we identified in only one or two replicates of ChIP experiments may be true targets of Arx and that the number of genes we selected as positive in all three replicates (1006 genes) may be slightly underestimated.

We then showed that quantitative expression levels of putative targets could be significantly altered by the ectopic expression of Arx in N2a cells or its knock-down in ventral telencephalon. In total, we found that approximately one quarter of Arx putative targets were deregulated following increasing or decreasing Arx levels in neuroblastoma cells or embryonic brain. However, this number is probably underestimated as Arx is normally not expressed in N2a cells and may thus lack binding partners and/or cofactors necessary to regulate the expression of certain genes. For example, we noticed that among the 159 ChIP-positive genes that showed gene expression changes in mutant brain, 135 genes did not show any mRNA change in Arx-transfected N2a cells. In addition, Fulp and Colasante have focused on ventral telencephalon for their gene expression experiments [21], [22], thus probably leaving out genes involved in neuroblast proliferation and/or radial migration. Similarly, as we selected only one stage of development in mouse in our ChIP experiments, it is likely that we missed several genes involved in earlier steps such as brain patterning or on the contrary later steps such as synaptogenesis and connectivity. Some changes in gene expression may also be too low to be detected in microarrays experiments. It is for example the case of *Gabrb3*, *Lmo3* or *Cdh2* that show a change of expression in qRT-PCR experiments following Arx expression in N2a cells (data not shown) but not on microarrays.

In conclusion, this study presents the first global analysis of Arx direct transcriptional targets. Identified genes are involved in a wide range of cellular processes suggesting new and expanded roles for this transcription factor. As detailed above, Arx directly regulates genes required for cell-cycle control, tight-junction dynamics, cell morphology, neuronal migration and differentiation as well as synaptic plasticity modulation, neurotransmission and axonal guidance. Among the different targets we identified, a number of genes are known to be important for brain development and some have already been linked to human disorders. But in addition, we identified new genes which may be good candidates to test in human neurological and psychological diseases. Further studies to understand their function and their relation to Arx will certainly bring new insight into the understanding of the pathophysiology of intellectual disability and epilepsy.

## Materials and Methods

### Cell culture and reagents

Neuro2a (N2a) mouse neuroblastoma cells (IFO50081) were obtained from the Japanese Collection of Research Bioresources (http://cellbank.nibio.go.jp/). These cells were grown in Dulbecco's modified Eagle medium (DMEM) high glucose (PAA) which was supplemented with 10% fetal bovine serum gold (PAA), 2 mM L-glutamine (Lonza), 0.1 mM non essential amino-acids (Gibco) and 2 mM penicillin/streptomycin (PAA). Cells were grown at 37°C with 5% CO2 and transfected with an expression vector expressing Arx using Lipofectamine 2000 according to the guidelines of the manufacturer (Invitrogen).

### Chromatin immunoprecipitation

Two days after transfection, 10^6^ N2a cells were fixed in 1% formaldehyde at room temperature for 10 min, followed by the addition of 125 mM glycine to stop the cross-linking reaction. Cells were then resuspended in ice-cold lysis buffer (50 mM Tris-HCl [pH 8.0], 10 mM EDTA, 1% SDS and Protease Inhibitor Cocktail (Roche)). To ensure that DNA fragments ranged from 300 to 600 base pairs (bp), samples were sonicated using a Transsonic T460/H (Elma) in an ice bath with 34 rounds of 30-s sonication pulses and an incubation of 1 min between each round. Samples were then centrifuged at 17 900 g at 4°C for 15 min to remove cell debris. Twenty microliters of the sonicated solution were stored at 4°C as Total Input, while the rest was incubated with 1 µg of either ARX C-terminal antibody (sc-48843, Santa Cruz) or anti-Arx-HD [15] or Pol II N-terminal antibody (sc-899, Santa Cruz) rotating overnight at 4°C. Immune complexes were captured the following day by addition of 40 µl of Dynabeads Protein G (Invitrogen) blocked with salmon sperm DNA (Applied Biosystems) and by incubation for 2 h at 4°C. Following washes with gradient stringent buffers, protein-DNA complexes were eluted by a solution of 1% SDS and 0.1 M NaHCO_3_ for 30 min at room temperature. The eluted solution, as well as the stored Input, was incubated with 0.2 M NaCl, 2 h at 65°C to reverse cross-link. Immunoprecipitated DNA and Input DNA were then isolated via phenol-chloroform extraction followed by ethanol precipitation. Concentration and purity of DNA were assessed using a NanoDrop spectrophotometer.

Chromatin immunoprecipitation on embryonic brains from wild-type mice was performed following a similar protocol. All animal procedures were performed in accordance with French and international guidelines and were approved by the French review board ministère (authorization ID: 29AUTEX005). Briefly, adult pregnant female mice were killed by cervical dislocation and brains were extracted from day 15.5 (E15.5) embryos in cold PBS. Following brain isolation, the whole forebrain including ventral telencephalon, thalamus, cerebral cortex and olfactory bulbs were frozen in liquid nitrogen and kept at −80°C for further experiments. For each experiment, tissue was pooled from 3 embryos. Tissue disaggregation and homogenization were performed in liquid nitrogen using a pestle and a mortar. Samples were then transferred into a 15 ml Falcon tube and fixed in a solution containing PBS, 1% formaldehyde and Protease Inhibitor Cocktail (Roche). Following steps were identical to those described above.

### DNA amplification, labelling and hybridization on microarrays

Equivalent amounts of immunoprecipitated chromatin and total input DNA were amplified in parallel, using a random primer method with the GenomePlex Complete Whole Genome Amplification Kit (15 cycles), according to the manufacturer's instructions (Sigma).

Two micrograms of amplified immunoprecipitated and input DNA samples were labelled with Alexa Fluor 647 or Alexa Fluor 555 respectively, by use of random primers provided in the BioPrime Plus Array CGH Indirect Genomic Labelling System (Invitrogen). A total of 5 µg of labelled DNA was then hybridised to mouse promoter ChIP-chip microarrays set (G4490A, Agilent Technologies) for 40 h at 65°C. After 4 successive washes, hybridization images were obtained using an Agilent DNA microarray scanner (G2505B) and intensity data were extracted using Feature Extraction software (FE v9.5.1) (Agilent Technologies). Results were visualised using the DNA Analytics software 4.0.85 (Agilent Technologies).

### ChIP-chip data analysis and positive probes selection

Intensity data on each array were normalized with the Lowess (Locally weighted scatterplot smoothing) method [34]–[36], pooled and represented on a graph. To identify regions of significant Arx association, the enrichment of each probe on the array was calculated as the log_2_-ratio of the intensities of Arx-immunoprecipitated DNA (y-axis) to control input chromatin (x-axis). One first important assumption is that points corresponding to non positive probes are distributed symmetrically around the axis x = y. A threshold spline curve was then defined by considering only the probes below this axis. Reporting this curve above the axis allowed discrimination between positive and negative probes. For each probe, a *P*-Value was obtained using a normalised distance between it and the axis x = y. All ChIP data are MIAME compliant and raw data have been deposited into NCBI Gene Expression Omnibus (GEO), and can be accessed with accession number GSE29985.

### Validation of target genes by PCR or QFM-PCR on ChIP-extracted DNA

Before DNA was applied to microarrays, the efficiency of chromatin immunoprecipitation was assessed by measuring the enrichment of *Ebf3*, *Lmo1* and *Shox2* promoters in Arx-immunoprecipitated material. Primers targeting these regions as well as the *Gapdh* control were the same as those published by Fulp et al. [21]. Real-time PCR was then performed using the Quantitect SYBR Green PCR Master mix (Qiagen) on a 7300 Real-Time PCR System (Applied Biosystems). The enrichment of the 3 targets was expressed as “% of Input” and was calculated following the SA Biosciences analysis instructions (www.sabiosciences.com/manuals/chipqpcranalysis.xls). Briefly, normalised Ct values for each sample were calculated by subtracting the Ct value obtained using input DNA from the Ct value obtained using Arx-immunoprecipitated DNA and the dilution factor [Ct(IP)] – [Ct(input) – Dilution factor]. Then, “% Input” was calculated using the formula: (2^−ΔCt^)×100.

Semi-quantitative fluorescent multiplex (QFM)-PCR was performed to confirm Arx fixation onto selected putative targets. The assay is based on multiplex PCR of short fragments and on the comparison of fluorescent patterns obtained from Arx-immunoprecipitated DNA and input DNA. Primers targeting Arx-bound regions were designed using PrimerQuest and Oligo Analyzer (IDT Integrated DNA Technologies, http://eu.idtdna.com/Home/Home.aspx) and are listed in Table S5. The optimal PCR conditions including primer concentration, annealing temperature, and number of amplification cycles were adjusted to ensure that each PCR was still in the exponential phase of amplification. PCR was performed with 100 ng of DNA, using the Multiplex PCR master mix 2× kit (Qiagen). Amplified DNA fragments were separated on an ABI PRISM 310 sequencer (Applied Biosystems) at 60°C using POP4 polymer and data were analysed by superimposing fluorescent profiles of immunoprecipitated DNA and input DNA using the GeneMapper v3.4 software (Applied Biosystems).

### Identification of motifs associated with Arx-binding

Motif discovery was performed on 450 bp masked sequences centered on the 500 best positive probes. We used the MDmodule of the MotifRegressor program [25], which allowed us to discover motifs of different sizes. In order to eliminate redundancies between all the identified motifs and compare them with Jaspar [37] and Transfac [38] databases, the MotifsComparator program [26] was used. Similar motifs were gathered leading to a consensus motif, which was generated with the WebLogo tool (http://weblogo.berkeley.edu/). A statistical significance for these motifs was performed using a Chi-square homogeneity test and comparing its score distribution in a set of positive probes and a set of non-positive probes.

Similarly, 400 bp-sequences flanking all positive probes were scanned using EMBOSS Profit (http://bioweb2.pasteur.fr/docs/EMBOSS/profit.html) for motifs corresponding to Prophecy-generated frequency matrixes of our discovered motif and the one defined by the study of Berger et al. [27].

### Luciferase reporter assays

N2a cells were transfected 24 h after plating in 24-well dishes with 500 ng of putative promoters cloned into the luciferase reporter plasmid pGL4.23 expressing the *firefly* luciferase (Promega), 200 ng of Arx-expressing vector or vector alone and 100 ng of pGL4.74 vector expressing the *Renilla* luciferase (Promega) using Lipofectamine 2000. Forty-eight hours after transfection, cell lysis and measurement of *firefly* and *Renilla* luciferase activity on a Fluoroskan Ascent FL microplate luminometer (Thermo Scientific) were performed using the Dual-Glo Luciferase Assay System (Promega) according to the manufacturer's instructions. Transfections were performed in triplicate. The *firefly* luciferase activity was normalized according to the corresponding *Renilla* luciferase activity to correct transfection efficiency and luciferase activity was reported as a mean relative to the results obtained with the expression vector alone.

### Functional annotations

Statistical analysis of the enrichment of Gene Ontology (GO) categories was performed using the Database for Annotation, Visualization and Integrated Discovery (DAVID) (http://david.abcc.ncifcrf.gov/) [39] and the Ingenuity Pathway Analysis (Ingenuity Systems) software (http://www.ingenuity.com/). Enrichment was determined in reference to the genes on the microarray.

### Gene expression analysis

Two days after transfection, RNA from N2a cells (1×10^6^) was isolated using the Total RNA Isolation Nucleospin RNA II kit following the manufacturer's instructions (Macherey-Nagel). After measuring RNA concentration using a Nanodrop ND-1000 spectrophotometer, RNA integrity was assessed with the Agilent 2100 BioAnalyzer (Agilent Technologies) by measuring the RIN. Samples were loaded on the RNA 6000 Nano chip. In addition, quantitative real-time one-step RT-PCR was performed to confirm the enrichment of *Arx* in samples transfected with Arx compared to samples transfected with plasmid control.

RNA (100 ng) was then labelled using the Low Input Quick Amp Labelling kit (Agilent Technologies). RNA spike-in controls were used to adjust possible dye effects. Briefly, 2 µg of total RNA were mixed with spikes-in and converted to cDNA using reverse transcriptase and oligo-dT primers in which T7 promoter sequences were added. T7 RNA polymerase was used for the synthesis and labelling of cRNA with either Cy3 dye or Cy5 dye. The fluorescent labelled cRNA probes were purified using the RNeasy Mini kit (Qiagen). An equal amount (825 ng) of Cy3 and Cy5 labeled cRNA probes were hybridized on Mouse Gene Expression 4×44 K V2 microarray (Agilent Technologies). Hybridization was performed for 17 h, rotating at 10 rpm at 65°C. Then, samples were washed and dried according to the manufacturer's instructions. Hybridization images were obtained using an Agilent DNA microarray scanner and intensity data was extracted using Feature Extraction software (Agilent Technologies). Transcriptomic data are MIAME compliant and raw data have been deposited into GEO and can be accessed with accession number GSE30190.

To characterize the differences in the profile of gene expression between control and cells transfected by *Arx*, the Lowess (Locally weighted scatterplot smoothing) method was used to normalize data expression [34]–[36]. Then, the matrix was filtered to eliminate low intensity probes and the ratio “*Arx* intensity/Control plasmid intensity” was calculated for each probe. Genes with differential expression were classified using the hierarchical clustering program Cluster [40] and the expression level of each gene was log-transformed. The clustering method used here was a centroid linkage with the uncentered correlation coefficient as similarity metric. Results were displayed using the Java TreeView program (http://jtreeview.sourceforge.net/) [41]. Information on probes was retrieved with the MADGene resource [42] and clusters of genes with the same regulation were then functionally annotated using GoMiner (http://discover.nci.nih.gov/gominer/index.jsp) [43].

### Gene expression analysis using Genespring software

To analyze the microarray data generated by Fulp and Colasante from E14.5 *Arx*-mutant brains [21], [22], we downloaded their datasets (GSE12609 and GSE12956) from Gene Expression Omnibus (GEO) and reanalyzed them using GeneSpring (Agilent technologies) for the statistical analysis. The method of Benjamini and Hochberg was applied for the multiple corrections with a *P*-value cut-off of 0.05 [44] and a fold change ≥1.1 or ≤−1.1 to obtain genes with a statistically significant change of expression.

## Supporting Information

Figure S1 Example of the expression of ChIP-positive genes representative of some of the enriched biological categories in E14.5 mouse brain. Images were obtained from the public database GenePaint. The majority of genes are expressed in the developing cortex and/or ganglionic eminences, consistent with a positive or negative regulation by Arx. Ctx: cortex, VZ: ventricular zone, CP: cortical plate, Hip: hippocampus, vTh: ventral thalamus, GE: ganglionic eminences, OB: olfactory bulb.
(TIF)

Table S1 ChIP-positive gene promoters identified only in N2a-transfected cells (sheet 2), only in embryonic brain (sheet 3) or in both (sheet 1). The indicated *P*-value for each gene corresponds to the geometrical mean of the best probe obtained from the 3 replicate experiments. Genes are listed in decreasing *P*-value rank order.
(XLS)

Table S2 Functional gene ontology categories enriched in ChIP-positive genes from N2a cells. The number and percentage of genes in each GO category are presented. The indicated *P*-value corresponds to modified Fisher Exact *P*-Value for gene-enrichment analysis (EASE score).
(XLS)

Table S3 Functional gene ontology categories enriched in ChIP-positive genes from mouse embryonic brain. The number and percentage of genes in each GO category are presented. The indicated *P*-value corresponds to modified Fisher Exact *P*-Value for gene-enrichment analysis (EASE score).
(XLS)

Table S4 List of ChIP-positive genes showing changes of expression following Arx overexpression in N2a cells and/or in *Arx* knock-out mice.
(XLS)

Table S5 Site-specific primers used in ChIP/QFM-PCR for the validation of Arx-bound promoter sequences.
(DOC)
