# Supplementary figures and images for: High-Throughput Analysis of Promoter Occupancy Reveals New Targets for Arx, a Gene Mutated in Mental Retardation and Interneuronopathies
Source: PLoS One. 2011 Sep 22;6(9):e25181. doi: 10.1371/journal.pone.0025181 (PMC3178625; doi:10.1371/journal.pone.0025181)

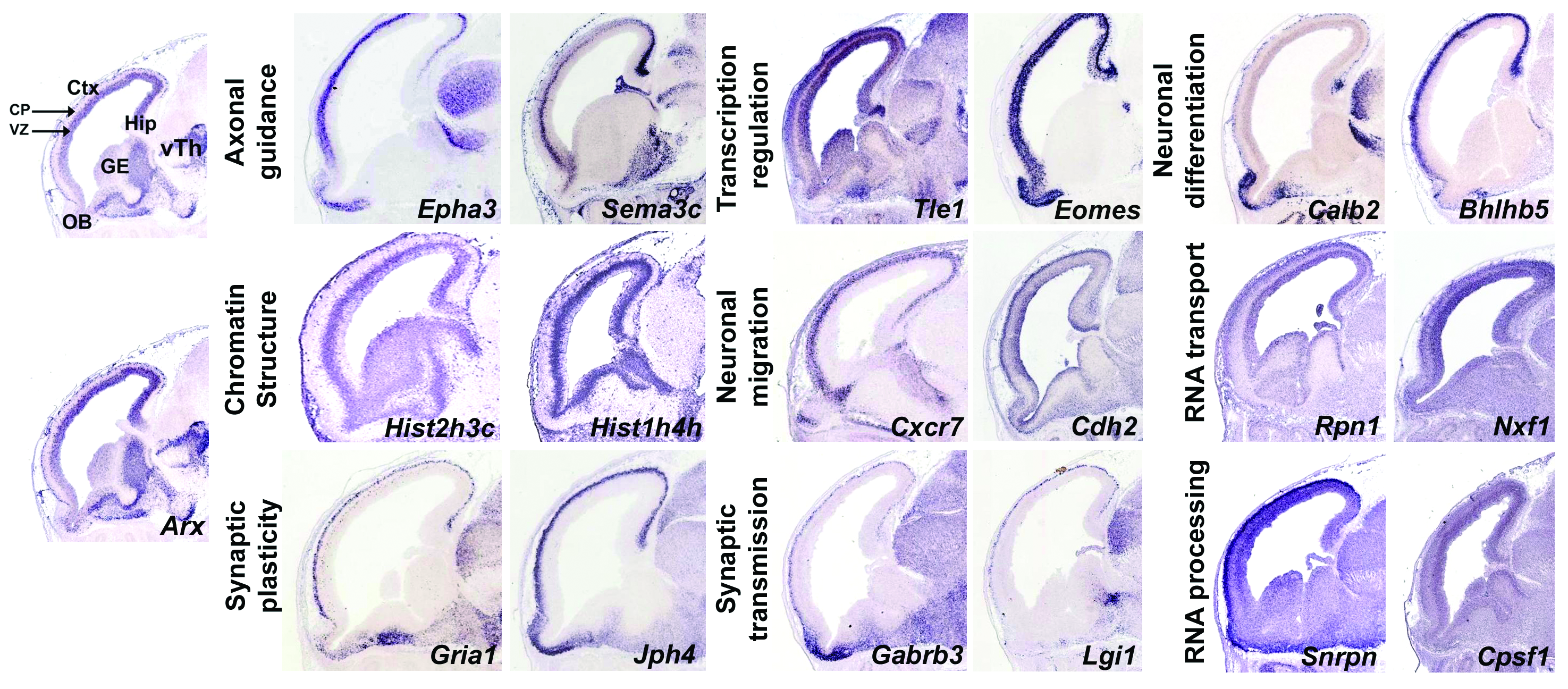

Supplement: Figure S1 — Example of the expression of ChIP-positive genes representative of some of the enriched biological categories in E14.5 mouse brain. Images were obtained from the public database GenePaint. The majority of genes are expressed in the developing cortex and/or ganglionic eminences, consistent with a positive or negative regulation by Arx. Ctx: cortex, VZ: ventricular zone, CP: cortical plate, Hip: hippocampus, vTh: ventral thalamus, GE: ganglionic eminences, OB: olfactory bulb. (TIF) [file pone.0025181.s001.tif]
