# Supplementary material for: High-Throughput Analysis of Promoter Occupancy Reveals New Targets for Arx, a Gene Mutated in Mental Retardation and Interneuronopathies
Source: PLoS One. 2011 Sep 22;6(9):e25181. doi: 10.1371/journal.pone.0025181 (PMC3178625; doi:10.1371/journal.pone.0025181)
Supplement: Table S5 — Site-specific primers used in ChIP/QFM-PCR for the validation of Arx-bound promoter sequences. (DOC) [file pone.0025181.s006.doc]

| **Gene** | **Forward primer** | **Reverse primer** |
| --- | --- | --- |
| Arid1a | GATCAAGGCATTCGCGGAAA | TTCCCTCTGGTTTCTCCTGG |
| Atrx | TTTACCTCATGGGCTCAGCG | TGCGGTGACTACTAAGATCCCA |
| Bhlhb5 | TTTGGAACGTGCCTATTGCG | TATCTGTTGGCCACAGGAACT |
| Calb2 | AGGTATTAGAAGTCCCTGCCTTGG | TAAACCTCCTACCCAAAGCCTTCC |
| Cdh2 | GGCAAAAGTCTGCCTTCAGGA | ACAAGAAGAAAGGGTGAGTGCC |
| Crb1 | CAGACTTGCATTGTTATCCTAGTCCA | ATTTAGTGCTCTCTGGTGTATTGGTT |
| Cxcr7 | TCAGCTAAGCAACCAGGGATTC | CCCAAGCTGGTCTCAAACTCAT |
| Ebf3 | CCGTAATGGATTTTGAGATGGGA | TGAATTGGTGGTGTGTGTGC |
| Epha3 | AGTGAGGTTAAGTTGACCATAAACG | TGTACGGACTACCCAGACAA |
| Gabrb3 | TCACATGGGCAAACTTGCTCTC | CACTCCACTCTAGCTCATGTCTGT |
| Gapdh | TACTCGCGGCTTTACGGG | TGGAACAGGGAGGAGCAGAGAGCA |
| Grm1 | CTTGCCTGCAAAGTGTCAGAAG | GCTGTTCGACATCCCACAAATC |
| Hist1h4h | ATGAGTTACCACTTGAGCGTCC | CGATGGATCGAAACCATCCTCT |
| Jph4 | TCAGTGTATCATGTGGGCAAGG | TCTGCTCTGATTGGATTCCAGG |
| Lmo1 | TAAGCTAATGGCGGGCACCT | CTCGCTCTCACCAGAGTGCA |
| Lmo3 | GACAGCAGATTTCTCATTTCAACTGC | AGTTCCCCTGTGTTTCCACAA |
| Olig3 | CCAAGGTCACCGTGCTAGTC | TCCGGAAGGAGTCAGTTCAGAT |
| Ppap2b | GTCCCGGCTGCATGTAAAC | ACAGTAGGCGCTCAACAAGT |
| Prph | CTTGGGCTCAGGTCTCAGA | AAGTTCCTACGGGACAGGAAAG |
| Pten | AAGGGCAGCAGCAACTTGTCTA | TGCCACTAATGTAATCCATCAAACCT |
| Sema3c | AACAACTGCTTTGCACTCGG | CTGCTGCAGGCTAGACTCTT |
| Sh3tc2 | ATTAGAGGTCTACTGTGCTCCCGA | AAGTGCCAGCCTTTAGAATGCG |
| Shox2 | TCCAGTTCCCCAGTGTTTTACTAAGT | GCTCTTGGCCATTAATCCAGGATT |
| Shroom3 | CTTTAAACCGAAGCCGACTTGAC | AGATAAACGAATCTCTCCGTGGG |
| Vapb | AGCTTGGCTCATCCTGTGAA | TTCGGTGATTGGCTCTGGAT |
| Zic3 | AGCTATTTGGCCGTGCAGA | TGCCGCATGTAACGGAAGAA |
